# Factors affecting recruitment and retention of nurses who deliver clinical research: A qualitative study

**DOI:** 10.1002/nop2.167

**Published:** 2018-06-25

**Authors:** Mary G. Boulton, Sally Beer

**Affiliations:** ^1^ Faculty of Health & Life Sciences Oxford Brookes University Oxford UK; ^2^ Oxford University Hospitals NHS Trust and Oxford Biomedical Research Centre University of Oxford Oxford UK

**Keywords:** nurse roles, nursing, qualitative approaches, research delivery, workforce issues

## Abstract

**Aim:**

To provide a better understanding of the factors affecting recruitment and retention of clinical research nurses.

**Design:**

Qualitative exploratory design.

**Methods:**

An on‐line questionnaire comprising open‐ended and fixed‐choice questions was completed by 121 clinical research nurses. Seven focus groups were held with a subgroup of 26 participants. Data were analysed using inductive thematic analysis.

**Results:**

Participants were attracted to a research nurse post by an interest in research itself, a desire for a change or to achieve personal objectives. The majority expected to continue in a research post for the next 5 years, while others expected to move on to research management, a clinical post or retirement; few had ambitions to become an independent researcher. Factors identified in focus groups as leading to intentions to leave research included desire for further change, concern about loss of clinical skills, rebalancing family/work responsibilities, short‐term contracts, unsupportive employers and limited career progression.

## 1 INTRODUCTION

Clinical research nurses, also known as clinical research co‐ordinators (CRCs), are widely recognized as fundamental to the success of clinical and applied health research (Larkin et al., 2012; Poston & Buescher, 2010). As skilled professionals, they are valued for the knowledge, experience and commitment they bring to delivering research in a wide range of settings (Fawcett & McCulloch, 2014; MacArthur, Hill, & Callister, 2014). In the context of a global shortage of nurses (Imison, 2015; Price, 2009; Rodrigo, 2013), however, recruiting and retaining nurses in research posts have become more difficult (Badger, Daly, & Clifford, 2012). Although clinical research posts are increasingly filled by those from other backgrounds (Boulton & Hopewell, 2017; Spilsbury et al., 2008), the importance of patient care and safety in the conduct of clinical research means that experienced nurses have remained in great demand. The continued growth of translational and applied health research, in the United Kingdom and internationally (Bell, 2009; Rickard & Roberts, 2008; Wilkes, Jackson, Miranda, & Watson, 2012), means this demand is likely to continue. To provide the means to meet it, a better understanding of what attracts nurses to and retains them in research posts is required.

### 1.1 Background

While several studies have described the rewards and frustrations of the research nurse role (e.g., Hill & McArthur, 2016; Rickard, Roberts, Foote, & McGrail, 2006, 2007; Rickard et al., 2011; Roberts, Rickard, Foote, & McGrail, 2006; Roberts, Eastwood, Raunow, Howe, & Rickard, 2011a, 2011b; Spilsbury et al., 2008), few have looked at what initially attracts nurses to a research post or what their current career intentions are. Published accounts of individual nurses suggest that their first research post was something they “fell into” rather than sought out and that their career in research was not consciously planned but evolved as opportunities arose over time (Grove, 2015; Sprinks, 2015). However, these are selected case examples which may not be representative of research nurses more generally.

Research on factors that affect retention of research nurses is also rare, though studies in clinical nursing are likely to have some relevance. In a study of three generations of nurses, for example, Robson and Robson (2015) found that intention to continue working in the NHS was associated with the combination of attachment to work, importance of work and (low) work–family conflict. Other studies have identified the following factors as associated with intention to remain in practice: perceived support from their organization (Masters & Liu, 2016), institutional initiatives to support their professional development (Bruyneel, Thoelen, Adriaenssens, & Sermeus, 2017; Duffield, Baldwin, Roche, & Wise, 2014; Kenny, Reeve, & Hall, 2016; Nowrouzi et al., 2015), a good work environment (Abou Hashish, 2017; Kenny et al., 2016; Laschinger, 2012) and success in or satisfaction with their career (Masters & Liu, 2016; Osuji, Uzoka, Aladi, & El‐Hussein, 2014). Factors associated with high staff turnover include exhausting workloads (Havaei, MacPhee, & Dahinten, 2016), difficulties in managing conflicting demands of work and family (Chen, Brown, Bowers, & Chang, 2015; Shacklock & Brunetto, 2012; Yamaguchi, Inoue, Harada, & Oike, 2016) and insufficient opportunities for professional and career development (Tummers, Groeneveld, & Lankhaar, 2013).

While these studies provide valuable insights regarding nursing in general, how they relate to research nurses in particular remains unclear. To address these limitations, a qualitative exploratory study was undertaken.

### 1.2 Aim

The aim of this paper is to provide a better understanding of the factors which affect recruitment to and retention of nurses in clinical research posts.

## 2 METHODS

### 2.1 Design

A qualitative exploratory design was adopted, drawing on qualitative elements of a larger mixed methods study which included other healthcare professionals (Boulton & Hopewell, 2017). The underpinning conceptual framework (Larson et al., 2013) sees recruitment and retention as dynamic social processes experienced by an individual in a wider social context. Key aspects of the social context affecting behaviour include motivations and satisfactions at the individual level; family responsibilities at the micro level; and policies and practices of employing organizations at the meso level.

### 2.2 Sample

Sampling and recruitment strategies are described fully in a previous publication (Boulton & Hopewell, 2017). Recruitment was through five NHS Hospital Trusts (including one University Hospital), two NHS Community Trusts and an NIHR Primary Care Partnership (11 GP practices) spread across three counties.

For the first stage of the study, a purposive “census” sampling strategy was used (Bryman, 2001). To be invited to take part in the study, all of the following inclusion criteria had to be met: nonmedical researcher, delivers (rather than initiates or leads) research, has direct contact with study participants, funded through an NIHR infrastructure organization, employed by an NHS Trust or GP practice and employing organization located in one of three counties of south‐east England. Exclusion criteria were Principal or Chief Investigator on a study or funded directly by a research grant.

A total of 280 individuals met the inclusion criteria and were sent an on‐line questionnaire and participant information sheet. Questionnaires were used to reach a large, diverse sample and to provide more information power for qualitative analysis, including simple counts and proportions (Malterud, Siersma, & Guassora, 2016; Maxwell, 2010).

For the second stage, a convenience sampling strategy was used to recruit a subsample to focus groups. An invitation was included at the end of the questionnaire; those who expressed interest were sent an information sheet and invited to choose from a list of dates and places for the focus groups. Focus groups were chosen to facilitate discussion and debate around professional issues of common interest to research nurses (Green & Thorogood, 2009; Kitzinger, 1994).

### 2.3 Data Collection

The questionnaire was developed by the first author on the basis of a literature review and an earlier unpublished study (Boulton, 2012). It comprised 56 multi‐part questions, including seven open‐ended questions and 49 fixed choice questions. Topics covered in the questionnaire relevant to this paper included Becoming involved in clinical research (one open‐ended question), Longer term career plans (one open‐ended question) and About you (six fixed‐choice questions). Questionnaires were completed anonymously; submission of a questionnaire was taken as consent to take part in the study.

As study participants were employed at NHS sites across three counties, it was important to hold several focus groups, each in a location convenient to their place of work, so that as many as possible could attend. Seven focus groups were held in five widely dispersed locations to ensure that research nurses from every employing organization could take part in one. They were held in Postgraduate Medical Education Centres or University meeting rooms and were facilitated by the first author, as numbers in each group were small.

Participants were told that the purpose of the focus group was to allow them the opportunity to elaborate on the topics covered in the questionnaire and to raise any further topics they felt important. Consent forms were signed before the start of each focus group. A topic guide was prepared and used flexibly. It covered several areas including what their post involved and how they felt about it, whether they saw research nursing as a long‐term career and why they might leave and what support they had received in developing their career. Questions were phased in terms of “Could you tell me about…” with additional prompts such as “What has been others’ experience?” Data collection started in June 2013 and was completed in January 2014.

### 2.4 Data analysis

As the study was largely exploratory in nature, an inductive thematic analysis approach was taken (Braun & Clarke, 2006). Responses to open‐ended questions on the questionnaire were saved in a spreadsheet, anonymized and read repeatedly by both authors. Initial codes were generated and revised following discussion by the authors. A final set of codes was agreed, the responses coded, the coded responses grouped into categories and categories grouped into higher order themes (Braun & Clarke, 2006).

Focus groups were audio‐recorded, transcribed, anonymized and read repeatedly. The process of qualitative analysis as described above was followed, including generating and revising initial codes, coding transcripts, grouping coded text into categories and categories into higher order themes.

Table 1 gives examples of the development of a theme from an open‐ended question in the questionnaire and a theme from transcripts of focus groups.

**Table 1:** Stages in analysis of qualitative data: Examples of codes, categories and themes

What attracted you to apply for your first post in clinical research?		
Examples of Codes	Categories (Sub‐themes)	Themes
Interest in research, BSc/MSc dissertation, previous research	Opportunity to pursue long‐standing interest in Research/research post	Research Focused
Create new knowledge, tackle health problems, evidence based practice	Attracted to research as a way to improve patient care	
Diversity, flexibility, variety	Attracted to specific aspects of research nurse role	
New challenge, something different, change direction	Looking for something new and different	Change Focused
New skills, better use of skills	Looking to develop new skills or use skills more fully	
Push factors, want to move, complete change	Looking to escape from current post	
Opportunity arose, spontaneous response to chance	Response to unexpected opportunity (Opportunistic)	
Invited to apply, offered post, head‐hunted	Response to invitation from someone else	
Career development, promotion, progression	Career advancement	Personal Objectives focused
Clinical area, research topic, consultant/clinical team	Opportunity to work in specific clinical area or clinical topic	
Mix clinical & research skills, keep patient contact in new context	Balance of clinical and research activities	
No shift work, off busy wards, family friendly hours	Better work conditions	

Factors affecting intention to leave research post		
Examples of Codes	Categories	Levels of Analysis
Explore other roles, lose interest in research, seek new challenges	Desire for further change in future	Individual level
Knowledge/skills out of date, struggle to go back to ward, risk drop in salary	Concern about loss of clinical skills	
Change in family responsibilities, less constrained, shift in focus/ambition	Rebalancing work & family responsibilities	Micro level
No job security, one year contract, unreliable employment,	Short term contracts	Meso level
Training needs not met, isolated & unsupported, skills/experience not recognised	Research and researchers not valued	
No career progression, no career pathway, not supported to progress	Lack of career structure & progression	

### 2.5 Rigour and trustworthiness

#### 2.5.1 Credibility

The authors are an experienced qualitative researcher who had conducted a previous study of research nurses and an experienced senior research nurse (Patton, 1999). Questionnaires were used to collect data from a large sample, and topics were explored further with a subsample in focus groups (Bryman, 2001; Green & Thorogood, 2009). Both authors independently coded the qualitative data and developed and refined categories and themes through iterative discussions (Braun & Clarke, 2006). All themes are supported by illustrative quotes.

#### 2.5.2 Transferability

Participants were recruited across a wide range organizations; response rates and sample sizes for both questionnaires and focus groups were good (Carlsen & Glenton, 2011; Morse, 2000).

#### 2.5.3 Confirmability

Key decisions in coding data and in refining categories and themes were noted, along with the rationale for them, to provide an audit trail (Bryman, 2001). All illustrative quotes are identified by a participant number (e.g., P138) or focus group number and transcript lines (e.g., FG2: 123–125).

#### 2.5.4 Dependability

Inclusion criteria and methods of sampling, data collection and analysis are described in detail (Shenton, 2004).

### 2.6 Ethical Considerations

The research protocol was approved by the University Research Ethics Committee. NHS Trust approvals were received from their R&D Departments. The work described in this paper was carried out in accordance with the Code of Ethics of the World Medical Association's Declaration of Helsinki (World Medical Association, 2001).

## 3 RESULTS

Completed questionnaires were received from 168 of the 280 individuals invited to take part in the wider study (Boulton & Hopewell, 2017), a response rate of 60%. Of these, 121 (72%) described themselves as nurses and constitute the questionnaire sample for this paper. Seven focus groups were conducted, ranging from 90 min to 2 hr and involving 26 individuals.

### 3.1 Sample characteristics

Table 2 shows the characteristics of both the questionnaire sample and the subsample who participated in focus groups. The characteristics of focus group participants reflect those of the larger questionnaire sample, with the exception of professional background: 19 (73%) were nurses, one (4%) was a midwife and six (23%) were other healthcare professionals.

**Table 2:** Characteristics of the questionnaire and focus group samples

	Main sample	Focus group sample
	(*N *=* *121)	(*N *=* *26)
Gender		
Female	116 (96%)	26 (100%)
Male	5 (4%)	0
Age		
<25	0	0
25–34	15 (12%)	6 (23%)
35–44	46 (38%)	7 (27%)
45–54	43 (36%)	9 (35%)
55–65	17 (14%)	4 (15%)
Qualifications		
Non‐degree (eg SEN, RGN)	40 (33%)	6 (23%)
BSc	53 (44%)	11 (42%)
PG Cert or Dip	8 (7%)	1 (4%)
MSc	18 (15%)	7 (27%)
PhD	2 (2%)	1 (4%)
Hours worked		
35+	61 (50%)	12 (46%)
20–34	42 (35%)	12 (46%)
19 or less	18 (15%)	2 (8%)
Years in current post		
Less than 1 year	26 (21%)	6 (23%)
1–2	37 (32%)	9 (35%)
3–5	42 (34%)	10 (38%)
6+	16 (13%)	1 (4%)
Years in all research posts		
Less than 1 year	8 (7%)	2 (8%)
1	26 (21%)	6 (23%)
2	17 (14%)	3 (12%)
3	13 (11%)	4 (15%)
4	14 (11%)	3 (12%)
5–9	25 (20%)	6 (23%)
10+	20 (16%)	2 (8%)

### 3.2 Initial attraction to research post

All 121 nurses responded to an open‐ended question on what attracted them to their first research nurse post. Three main themes—research focused, change focused and personal objectives focused—were identified in their responses.

#### 3.2.1 Research focused

Almost a third (37, 31%) of participants indicated that an interest in research itself was the primary attraction of a research post. For these participants, it was the opportunity to be involved in research that was important to them. Most had a longstanding interest in research, often deriving from their undergraduate or postgraduate degree and saw a research nurse post as providing an opportunity to pursue this interest further:

I have wanted to work in clinical research since completing my BSc dissertation (P71)

Always was interested in research (P114)

Some were interested in research as a way of improving patient care through the creation of new knowledge and evidence‐based practice. A post as research nurse was seen as a way of achieving this:

Wanting to contribute to furthering the knowledge of and treatments for xxx (P69)

Others were attracted by what they perceived as key features of the research nurse role, including “flexibility/variety in research” (P93) and “diversity of the role”. (P160)

#### 3.2.2 Change focused

A larger proportion of participants, 45 (37%), indicated that what had attracted them was a change from their current post. For these participants, it was not research itself but the opportunity to do something new and different that was important:

I wanted a change in my career. (P22)

For most, a research post was seen as offering a “new challenge, something different from previous role (P124)”or “an exciting opportunity (P55).” Others saw research as offering a chance to develop new skills or use skills more fully:

Expand my skills in a disease area which I had a lot of clinical experience in (P97)

For several participants their route to a research nurse post had been largely opportunistic: they were looking for a change and a research post came up:

It was a secondment opportunity in my department which I applied for simply for a change in daily duties, not really that I was interested in research nursing. (P31)

Other participants had been invited or encouraged to apply for a research post by someone else. In contrast to the previous group, they had not actively looked for change, but had accepted it when offered:

Further to a personal invitation from the Project lead… (P146)

For still others, the driving force behind their move was a desire to leave their current post:

I wanted to come away from ward work because I was suffering from neck and shoulder pain related to the work. (P32)

#### 3.2.3 Personal objectives focused

About a third of participants (39, 32%) indicated that they were primarily concerned with personal objectives which were tangential to research, but which could be achieved through a research post. The first objective identified was that of career advancement, though not necessarily in a research career:

I had been unsuccessful in applying for more senior nursing positions and feedback from interviewers was that the successful applicants had research experience which was looked on favourably so I started looking for research posts to “pad” my resume. (P107)

A second objective was to work in a specific clinical area or clinical topic which a research nurse post made possible:

The post was for a xxx Research Nurse and I was really interested in working in [clinical area] for the first time in my career. (P64)

A third goal was to achieve a better balance of clinical and research activities, or retain contact with patients:

The opportunity to still have patient contact, but to do something totally different. (P164)

Finally, for some participants, better work conditions or a better fit with family responsibilities were particularly important objectives and a research post was seen as a way to achieve them:

I was a return‐to‐practice nurse graduate… I tried working in the ward setting but the 12 hr shifts and a young family made it impossible for me. Research nursing gave me the chance to use all my previous skills and expertise and fit with my family. (P135)

### 3.3 Five‐year career intentions

A total of 110 nurses responded to an open‐ended question on what ideally they would like to be doing in 5‐years time. Four main themes—continue in research nurse post, progress towards independent researcher, leave research and uncertain—were identified.

#### 3.3.1 Continue in a research nurse post

The majority of those who responded (67, 61%) indicated that they wanted to continue in a research post. Almost half of these indicated that they would like to continue at more or less the same level as they were, possibly with some change in focus or a greater variety of studies:

More of the same, with more trials open which vary in type, e.g., Phase 1‐4. (P90)

Almost as many wanted to continue as a research nurse but with some sense of career progression, for example, in a senior research nurse post, taking on more responsibility or leading a team of researchers*:*

Working more hours in a similar, or possibly more senior role, i.e., Senior research nurse and possibly in a more clinical environment. (P62)

#### 3.3.2 Progress towards nurse researcher

A much smaller proportion (11, 10%) indicated that they wanted to take steps consistent with becoming an independent researcher. Some phrased this in terms of gaining a higher degree:

Undertaking a PhD. (P22)

Others in terms of carrying out their own research:

Developing my own research study… (P50)

Only two, however, explicitly stated that they wanted to “Become a nurse researcher (P76).”

#### 3.3.3 Leave research

A fifth of participants (21, 19%) would like to move on from a research nurse role to something else. For some, this meant a move into research management or governance:

Working in an R&D department supporting researchers. (P71)

For others this meant a move back into a clinical role, generally at a higher professional level:

Ideally I would be working closely with patients—CNS [clinical nurse specialist] for xxx would be my ideal post. (P81)

And for others it meant leaving nursing altogether, either to a job outside nursing or to retirement:

Owning my own teashop (P18)

Most likely I will be retired within the next 5 years (P146)

#### 3.3.4 Uncertain

The last group comprised those (11, 10%) who wanted to remain employed but were uncertain as to what they would like to be doing in 5‐year time. This included both experienced nurses:

At this point I'm really not sure (P44)

and those new to nursing:

I need to understand what other roles are available first. (P151)

### 3.4 Factors affecting intentions to remain in or leave research nursing

Views, concerns and experiences underpinning intentions to remain in or leave research nursing were discussed in the seven focus groups. These are grouped in terms of factors operating at individual, family (micro) and organizational (meso) levels, all of which could change over time.

#### 3.4.1 Individual level

Almost all participants expressed enjoyment and satisfaction in their roles as research nurses. The interest and variety of their work, rewarding interaction with patients, greater autonomy and new opportunities to learn and develop all contributed to intentions to remain in research nursing:

I like the practicalities, I love interactions with patients and… this is to me as good a career as I can get. (FG3: 830–832)

However, not all participants were certain that they would continue to find research nursing satisfying over the longer term:

I've got another twenty odd years of nursing to go—do I necessarily want to be doing this for 20 years?… I don't know, because I think there are lots of other things to explore out there…. I like a bit of variety and I might fancy something different 5 years down the line. (FG6: 1041–1044)

Related to this was a concern that, if they remained in a research post “too long,” their clinical skills would decline and so limit their ability to return to clinical practice in the future:

A: My clinical skills have certainly taken a nose dive….

B: I came from a xxxx background and I couldn't go back there because it's moved on.

C:… if I went back into clinical practice, I'd have to go in at [lower pay band] (FG1: 1556–1579)

For these latter participants, a quest for change and new challenges or their concern to keep job options open may result in their departure from research nursing at some point in the future.

#### 3.4.2 Family level

Focus group discussions also provided an insight into the way family responsibilities framed participants’ decisions when considering a research nurse post. Family friendly hours and more bounded responsibilities were significant attractions for some participants and, while their children were young, helped to maintain their intention to remain:

At the moment I do what I do because it fits in with my family life. I've got young children and that works very well. (FG1: 1408‐ 1409)

However, as children grow up and become more independent, these features provide less of an incentive to remain in a research nurse post and, unless other factors such as intrinsic satisfaction come into play, at least some are likely to consider moving on to another role. As the previous participant went on to say:

In another 5 or 10 years, when my children are older and I want more of a career again, I don't know whether it will hold enough for me. (FG1: 1409‐1411)

And as another stated:

I'm coasting. I've got a young family at the moment, so this is what [I am focusing on] but no, that's where I'm heading, definitely [out of research]. (FG4: 2629‐2630)

#### 3.4.3 Organizational level

A range of policies and practices in their employing NHS Trusts were also raised as contributing to participants’ intentions both to leave and to remain in research nursing.

Short‐term contracts were the most common cause of concern. Over 90% of participants had a contract of employment but for most this was for 12 months or less. The uncertainty and insecurity this caused raised questions about the viability of a research nurse post as a long‐term option:

I know the job insecurity gets to an awful lot of people… [Two] of my colleagues have left recently precisely for that reason. If I have a family I don't want to be thinking every year, “Oh [goodness], what am I going to do?” So that might make me leave, where I don't want to, but I might leave just because of that. [FG5: 1523‐29]

A further concern for some was a sense that they and their work were not adequately valued by their NHS Trust, as reflected in, for example, assumptions among managers “that anyone can do it [research]” or that it was acceptable for clinicians to take over their clinic room because “it's research, it's not real patients” [FG3: 2286–2298]. It was also evident in the perceived lack of support for personal or professional development. For example, participants discussed how their efforts at professional development had been resisted:

R: I would like to build up… [to] becoming a senior research nurse, leading a team of nurses and things, but from an IPR [Individual Performance Review] point of view, when I brought that up, it's not backed. So basically, I don't think there's any scope for it here at the moment.

S: No, they don't want that, I had that feeling as well. I don't feel that they want us to develop more…. [FG7: 731‐737]

Such experiences could be demoralizing and foster an intention to leave.

Similar frustrations were expressed in relation to limited opportunities for career progression, partly attributed to a lack of career structure:

The only issue I have is that at the moment there isn't career progression… [There] have been a lot of grumbles from different research staff that there is no progression for a research nurse… [If there is no prospect of career progression] then I would probably seriously consider going over to the other side of the fence into industry [FG4: 2224–2240]

In contrast to these negative aspects, three other features in their employing NHS Trusts were identified as supporting intentions to remain. The first two were supportive mentors and internships which helped to ensure that those nurses new to research got a good grounding in the practical knowledge and skills needed to succeed in their careers:

[We're] in the process of developing a mentorship scheme for that to happen and looking very much at developing the early career path for research nurses [FG1: 236–237]

So maybe with the development of this internship, then people might come and decide that research is a place that they want to be and progress right through to nurse researcher. [FG1: 1488–1489]

The third was working in an NHS Trust with a large and lively research environment which could provide help for research nurses to develop “academically” [FG3: 840] and the “right environment” in which to progress [FG3: 869]. This was particularly evident in relation to NHS Trusts associated with a research intensive university which provided longer term prospects and a wider range of opportunities for researchers:

I'm going to be in it for the duration. The fact I'm on a 2‐year rolling contract is maybe putting some people off… But if you're making a good job of things, then somebody else will find you the next project to roll on your contract. [FG6: 571‐ 74]

This continuity of employment and at least the potential for career progression in research gave these nurses the confidence that underpinned their intention to remain in research over the longer term.

## 4 DISCUSSION

This paper has explored what attracts nurses to and retains them in a research nurse post using qualitative methods and a conceptual framework which sees recruitment and retention as dynamic social processes experienced by individuals in a wider social context. Table 3 shows the factors that were found to affect this process at the individual, family and organizational levels.

**Table 3:** Factors affecting recruitment and retention of research nurses

Time (changing circumstances and priorities) ——————→———————————————————→		
Recruitment to research nurse post	Current intentions to remain in research nurse post	Longer term intentions to leave research nurse post
**Individual Level** Initial attractions/motivations: Research focused Change focused Personal objectives focused	**Individual Level** Satisfaction & Enjoyment: Interaction with patients Using skills more fully Autonomy	**Individual Level** Thinking of the future Desire for variety, change Looking for new challenges Concern over loss of skills
	**Micro Level** Fit with family responsibilities	**Micro Level** Rebalancing family and work responsibilities
	**Meso Level** Internships Supportive mentors Large/lively research environment	**Meso Level** Short term contracts Research &/or researchers not valued Lack of career structure & progression

In terms of recruitment, it is striking that only a minority reported an interest in research *per se* as an attraction, though this is, perhaps, not surprising given the limited experience of research that nurses gain at pre‐registration level (Deave, 2005; Badger et al., 2012; Loke, Laurenson, & Lee, 2014). For most participants, the attractions lay in other features of the post: the change or new challenge it offered provided “job enrichment” (Duffield et al., 2014); family friendly hours helped in managing conflicting demands of work and family (Powell & Greenhaus, 2010); and other features enabled the achievement of a range of personal goals, from working in a specific clinical area to gaining promotion in clinical practice (Duffield & Franks, 2002).

As in clinical nursing, the variety of attractions described may also reflect the range of career stages and personal circumstances of the study sample. For example, Pool, Poell, Berings, and ten Cate (2015) found that orientations to work changed over time in ways that resonate with those identified in this study, for example, learning more about research and using their skills more fully; seeking new challenges or a better work/life balance; and working in their chosen clinical field.

While studies in clinical nursing suggest that career planning is an important element in achieving career progression (e.g., Donner & Wheeler, 2001; Hall, Waddell, Donner, & Wheeler, 2004; Shermont, Krepcio, & Murphy, 2009), there was little evidence of career planning in participants’ accounts. This is consistent with an Australian study of research nurses which observed that most participants had not planned a career in research (Rickard et al., 2011) and with published accounts of individual research nurses in the United Kingdom (Grove, 2015; Sprinks, 2015).

In terms of retention, the majority of participants reported that they would like to be still working in clinical research in 5‐year time, reflecting the interest and satisfaction they currently derived from their roles (Rickard et al., 2007; Roberts et al., 2006; Rodrigo, 2013). However, as became clear in the focus groups, this did not mean a commitment to remain in research indefinitely: the possibility of a return to clinical work when their children were older was a recurrent theme and is evident in the short time (median 3 years) participants had worked as a research nurse. Similar findings have been reported in other studies: Rickard et al. (2006) reported 5 years as the median time that nurses indicated they would continue in a research role; Wilkes et al. (2012) reported 8 years as the mean time nurses had worked as clinical trials nurses; and Jeong (2012) reported that less than a third of Clinical Research Co‐ordinators had remained in post for more than 3 years.

Only two participants explicitly stated that they would like to become a nurse researcher. This suggests that research nurses rarely view “nurse researcher” as their career ambition but may instead look for career progression in research nursing itself. In the United Kingdom, several attempts have been made to address this issue, with documents specifying key competencies for clinical research nurses at each of four pay bands and so “[providing a] clear structure for career development” (Competency Working Group, 2011: 6). Similar initiatives can be seen in the United States (NIH Clinical Centre, 2017). However, as discussions in the focus groups demonstrated, lack of career progression remains an important contributor to intentions to leave research nursing.

Short‐term contracts and lack of institutional support for career development were also identified, in this and previous studies, as contributing to intention to leave (MacArthur et al., 2014; Rickard et al., 2011; Rodrigo, 2013). To these factors, we have added concerns about losing their clinical skills (and future job prospects) which are likely to remain until there is greater scope for career progression in research nursing.

This study also supports findings from clinical nursing of the importance of mentors and internships (Byrne, Topping, Kendall, & Golding, 2014; Cleary, Sayers, & Watson, 2016; Price, 2009). The importance of a lively research environment as enabling research nurses to flourish and progress is another novel contribution of the current study. The continuity of employment offered by a large research active institution provides research nurses the opportunity to build experience and expertise. Their role in “the team” also affords a good basis for career progression. Outside research intensive centres, however, jobs may feel less secure and opportunities to progress less evident. In these circumstances, a move to more administrative posts such as trial manager or into research governance may be their only options.

### 4.1 Limitations and implications for further research

Research nurses who were employed by NHS Trusts and GP practices to deliver research but were not funded through NIHR infrastructure facilities were excluded from this study as it was not possible to identify the relevant population. This limits the generalizability of the study findings and further research is needed to understand how other systems of employment, particularly the employment of research nurses as research assistants working as members of the research team and funded directly by a research grant, affect recruitment and retention. The study is cross‐sectional and, while it has identified several factors which, if addressed, could help to improve recruitment and retention of research nurses, further longitudinal work is needed to test specific hypotheses. All participants were employed as research nurses at the time of the study and their discussion of the reasons for leaving research nursing were well informed but speculative. Further qualitative research is needed with a sample of nurses who had recently left a research nurse post to provide a better understanding of the circumstances when they leave and where they go when they have left.

### 4.2 Implications for policy and practice

Challenges to recruiting and retaining sufficient research nurses to meet increasing demand internationally will need to be addressed in several ways.

#### 4.2.1 To address recruitment issues

At undergraduate level, an interest in research could be encouraged by providing more opportunities for nursing students to experience research through placements with research nurses and through conducting primary research for their dissertation (Loke et al., 2014). Support for new graduates in career planning (Byrne et al., 2014) and internships or mentoring schemes for those new to clinical research (Cleary et al., 2016; Jones‐Berry 2016) are also important in attracting and retaining new nurses. To recruit experienced nurses, other nurses or health professionals could act as “research advocates,” identifying talented nurses and encouraging them to take up a research post, while recognizing and supporting their diverse motivations for doing so.

#### 4.2.2 To address retention issues

To retain experienced research nurses, it will be essential for employing organizations to address policies and practices which create insecurity, appear to demean their work or dismiss their ambitions for professional development and career progression. Fostering networks of research nurses within and across research sites, including university hospitals, may also provide greater opportunities to access a wider range of research posts and greater potential for progressing in their research careers. Nursing organizations such as the Royal College of Nursing also have a role to play in supporting “the professional recognition and status that researchers in other settings are afforded” (Rickard et al., 2011: 166) and in turning policy documents on a career in clinical research into reality, by supporting the progression of research nurses through to equivalent of Clinical Nurse Specialist, Advanced Practitioners and Nurse Consultants.

## 5 CONCLUSIONS

The factors affecting recruitment to and retention of nurses in research posts derive from the motivations and family responsibilities of individual nurses, the policies and practices of employing organizations and how these change over time. Most nurses in a research nurse post are attracted by aspects of the role that meet their interests and concerns at the time. As these change and as the frustrations consequent to the policies and practices of their employing organization become more salient, many leave research for a clinical or administrative post that better meets their new interests and circumstances. This suggests that retention will continue to be a problem until key institutional disincentives are addressed. While specific aspects of the research nurse role may continue to attract new applicants and so replenish the workforce, recruitment of nurses with an interest in pursuing a long‐term career in research may also continue to be problematic. A range of initiatives are needed in both educational and employing institutions to address these challenges.

## CONFLICT OF INTEREST

No conflict of interest has been declared by the authors.

## AUTHOR CONTRIBUTIONS

Mary Boulton conceived and designed the study, led on analysis and wrote the first draft of the paper. Sally Beer made a substantial contribution to the analysis and interpretation of the data. Both authors have given approval for publication and agreed to be accountable for all aspects of the work.
